# Harvard Odontological Society

**Published:** 1902-06

**Authors:** 

**Affiliations:** Harvard Odontological Society


### HARVARD ODONTOLOGICAL SOCIETY.

THE regular monthly meeting of the Harvard Odontological Society was held November 21, 1901, at Young's Hotel, Dr. Waldo E. Boardman presiding in the absence of the President.

An address was given by Dr. E. A. Crockett, of Boston, subject, "Deaf Mutes."

(For Dr. Crockett's address, see page 405.)

### DISCUSSION.

*The President.*—This interesting subject is now open for consideration. I will ask Dr. Werner if he will open the discussion.

*Dr. Werner.*—I do not see how I can open a discussion on a subject upon which I feel so totally ignorant, and in regard to which, so to speak, I have heard my first talk. I very frequently see these deaf mutes that Dr. Crockett has examined in the Horace Mann School, and I pity them; most of us do. And I pity them more now, when I think how many of them might be in much better condition than they are.

I was in hopes that the doctor, in some way or another, would tell us of the possible relation between the teeth and the ear, and I hope he will yet tell us that. We are very much interested to know how any dental trouble might be, by neglect or maltreatment, conducive to deafness. In that direction I am sure we are all very much interested.

Another thing that was very suggestive to us all, no doubt, was the question of adenoid growths. I think the time is coming when we as dentists will have much more to treat in that direction. In my own family I have had both my children treated, and it certainly relieved what seemed like a good deal of earache and the tendency to mouth-breathing, and the operation seemed justifiable.

The suggestion is in the direction of how many of these deaf mutes in our public schools, the Horace Mann School, and the Northampton Institute (something like fifty per cent.) are due to diseases that could be cured, principally adenoid growths; and when we know the condition of our climate, and that of Norway, Sweden, and the northern parts of Germany, where we find deaf mutes are prevalent, we can see that we as dentists will eventually have that question come more or less under our special treatment. Many of our little patients come to our hands at an age when it is decidedly to the patient's advantage, and decidedly our duty, that we find out whether they have adenoid growths or not. I think likely there is no other body of specialists that would be half so intelligent on the subject as the dentists, or that would have half so good a chance. The child has measles or bowel troubles, and the general practitioner does not examine its mouth or its nose; but when that little patient comes to our hands, the mouth is examined, and we might take a step farther and examine the nasal posterior region, and decide the question of whether adenoids are present or not. In my own practice it has been many times suggestive, at least, of having the patient treated by special or competent hands; and perhaps if I had not made a suggestion it would have been allowed to go on, and there would have been more serious trouble.

*Dr. Naylor.*—There is just one question, in regard to teaching the scholar the method of lip understanding,—learning what a person is saying by watching the lip, and at the same time the child itself learning to talk. I would like to know whether, as a general rule, the sound of the patient's voice is similar to the sound of the ordinary person's voice when they are learning to talk?

*Dr. Crockett.*—If the patient has hearing enough to get a perception of sound in the way I spoke of, by putting the hands on the piano and getting an idea of pitch, then their voice will be natural after from three to five years of education.

*Dr. Naylor.*—Why I ask that question is that I have a patient that was born without being able to hear or speak, and she has learned the method of lip understanding so that she can readily understand anything; but in talking there is a very peculiar sound to the voice: it is not the sound of an ordinary person speaking. It seems to be somewhere way back in the head, and it takes a certain amount of concentration on the part of the listener to understand what she says. I wondered whether that was the ordinary case with these patients or not.

*Dr. Crockett.*—That is the general case, up to about five years of education; and it is the universal rule where they possess no hearing whatever, so that they have no idea of pitch. I took a number of the children from there who talked in that way, and who have been educated for five or six years, and removed the adenoids, and it improved the resonance of the voice in a number of cases. Of course, if they have no conception of sound or pitch, they cannot tell whether their voice is loud or soft, or whether it is in their heads or thrown out.

*Dr. Cooke.*—I would like to ask the doctor at what age an examination would amount to anything,—*i.e.*, to give good treatment to prevent these patients, this fifty per cent., from being deaf on account of the adenoid growths? That is, when the children go to school, at four years of age, or when they enter the primary school or the kindergarten, if they were examined then, would that be too late?

*Dr. Crockett.*—No, I think that would strike nearly every case.

*Dr. Cooke.*—Then if we had a system of examinations in our schools in regard to the physical condition, it would cure a very large amount of troubles. Then in connection with the eye troubles, with blindness, does not a large share of that come, as with deafness, from a lack of treatment?

We are in the same fix in regard to dentistry. The patients do not come to us until they have had trouble. And if we are going to make a success in the future, our victories must be won before the child is ten or twelve years of age. But we are at a disadvantage, alike with these men who are treating these other troubles. If the children could be examined who go to school, then their parents would understand, from a reliable source, just what condition the child is in physically. I hope we shall see the time when every single child that enters our public schools shall have that kind of an examination, so that the systems of both men and women shall have at least good bodies to begin on, and bodies that have been passed on by competent authority so that these things can be cured.

*Dr. Werner.*—May I ask to what extent does a public physician, assigned to a school, as I understand is the case in Boston, render any real service to the individual child, except in the case of epidemics?

*Dr. Crockett.*—I do not want to criticise the system, but there is a physician to every two or three schools in Boston, and so far as I have seen the examinations made, the physician will simply go in and spend fifteen minutes in a room of fifty pupils, and look them over, and then he goes out and goes to the next room.

*Dr. Bigelow.*—Is it not true, Dr. Crockett, that the parents interfere with such medical examination somewhat, and make it difficult for the inspectors to do their work?

*Dr. Crockett.*—I do not know. They made it rather awkward for me in another way. A great many of the children had been seen by other physicians, and I found the parents were immensely interested in the process. They would come around personally to the school: none of them objected to the examination, and no one objected to any operation I thought wise. I think there is a great difficulty that you may interfere with some other man's treatment; you must be very careful about that, that you do not take the case away from any man in private practice.

*Dr. Bigelow.*—Is this medical inspection done as a gratuitous service?

*Dr. Crockett.*—I understand they pay a small salary.

*Dr. Giblin.*—I think it is very important that we as dentists, who come in contact with so many children in our practice, should be able to direct them to specialists in the care of the ear and throat; and it occurred to me in connection with a little girl I have in charge now, who is partially deaf. She has reached the age of twelve or thirteen years. She had a lesion or an abscess form in one ear, and she is quite noticeably deaf. I suppose it has reached the limit when it is past curative treatment, judging from what Dr. Crockett said; but personally I should like to be able to direct the parents, if it is not to late, to attempt some treatment for the case. She was operated on for adenoids, and subsequently there was an abscess formed in the right ear. She is attending the public schools, and has reached the graduating class in the grammar school. She is quite bright. Is that too late to undertake treatment?

*Dr. Crockett.*—That is a very difficult question to answer. I

think the chances are in deafness as in everything else, as if she had a broken arm eight years ago, and it was now attempted to set it: the chances are it is difficult to do anything with it. It is possible it might be improved. I doubt very much if after seven or eight years her hearing could be made normal. I do not believe she would have one chance in fifty of obtaining a perfect ear or anything like it. When it comes to adult life you cannot do much in the way of treatment.

*Dr. Giblin.*—Does a lesion in one ear always affect both?

*Dr. Crockett.*—No, not at all.

*Dr. Giblin.*—This child is deaf in both ears.

*Dr. Grant.*—I would like to ask Dr. Crockett a few questions, and one is regarding what he said about Sweden and Norway. I am in the condition Dr. Werner is in: I never heard anything about it before. I should like to know whether the deafness is a result of climatic causes entirely?

*Dr. Crockett.*—It is very much more common in northern countries, and especially in countries like Denmark. The percentage is large there, because they have a very changeable climate and are entirely surrounded by water, so they get moisture as well as cold. They have a Boston climate in every part of Denmark, and they have a great deal of trouble in that country.

*Dr. Grant.*—Another thought: when you were speaking of the increase in the number of deaf mutes from intermarriage, I wondered how much that could be due to the immigration of people from those countries. In the last fifteen years there has been a very largely increased immigration from those countries. I wonder whether some of it might not be due to that.

*Dr. Crockett.*—I suppose very likely. I could tell with these children because I have the birthplaces of all of them.

*Dr. Cooke.*—I should like to ask one more question. I understand that there is on Martha's Vineyard Island quite a community of these deaf mutes who have married and intermarried.

*Dr. Crockett.*—I had never heard of that.

*Dr. Cooke.*—There are quite a number of them there. I wonder if the statistics refer to these.

*Dr. Crockett.*—I would not want to be construed as criticising the present method of school examination: I think the present method is all right, except that it does not go anywhere near far enough. The examiner is not paid enough for the examinations to make it any object to him. I do not know exactly what it is, but I think two hundred dollars a year, or thereabouts, for examining two or three schools at a time, and making the examinations once or twice a week. It does not make it an incentive to a man. He is not expected to go much beyond contagious diseases. I think, as the doctor stated, he should examine the teeth and the eyes and the ears, which are as important as to find out whether they have got measles, and a good deal more so.

*Dr. Grant.*—I would like to ask one question, perhaps not especially relative to deaf people; but I have heard it stated a great many times that the ear of the female was able to hear a greater number of vibrations that that of the male.

*Dr. Crockett.*—No, I do not think that is true. I think it might possibly be true in this way, that the ear could be trained to a very considerable extent, and possibly more women are versed in music. naturally, I think, when they are born, for instance, there is no difference.

*Dr. Grant.*—I have heard it a great deal. It is really taught in some music schools as a fact, and I never could quite reconcile it to my ideas of the fitness of things. They claim, for instance, that a woman or a girl will hear a higher pitch note; that is, after it has gone beyond a man's hearing, a woman can still hear it.

*Dr. Crockett.*—I do not believe you could substantiate that scientifically. If a man is musically educated his auditory nerves are very much keener.

*Dr. Gilpin.*—I should like to have Dr. Crockett answer Dr. Werner's question,—if he has ever had any experience of deafness from dental irritation? or is there such a possibility?

*Dr. Crockett.*—I do not think there is. I think you get a good deal of neuralgia in the ear, but I do not believe you could get an actual deafness from purely dental irritation. Of course you might from an abscess, or something of that kind; but otherwise not.

*Dr. Werner.*—Could we not through an impacted tooth, or a badly situated or abnormally situated third molar? Of course, you would in all those cases, would you not?

*Dr. Crockett.*—I do not think I ever saw one that I thought came directly that way. I know some of the text-books say it does.

*Dr. Boardman.*—The chair will state in connection with this that the Massachusetts Dental Society, at its last meeting, took into consideration the question of appointing a committee to examine the school children of Boston and Massachusetts, and the subject is now before them, and at the next meeting they expect to report as to what they have done and the methods of procedure in that line. They expect to proceed through the Board of Health, I understand, and examine all the children's mouths that they can.

*Dr. Werner.*—Just one suggestion. If it comes to that universal examination of our children in the public schools, I have two children who are both going, or have been, through public schools, and I hope they will have something like thorough asepticism practised. I should most decidedly object to having my child examined with the same mouth mirror used for some one else, and exposed to contagious lesions possibly. It opens a very wide field, and if our Dental Society suggests anything in that direction they must be careful in these particulars. We are just waking up thoroughly in our profession and in our schools, etc., to the care we should give ourselves, as well as preventing contagious diseases. I think that is the universal feeling among the children,—a feeling that great harm may be done in that direction. Intelligent people would not object to it if they could be convinced that it is thoroughly up to date.

*Dr. Moffatt.*—The secretary will remind the Society that the National Dental Association have invited us to co-operate with them on the subject of oral hygiene in our public schools. A letter was read last May, and nothing has yet been done about it. There is an opportunity for those who are interested to start the good work.

*Dr. Baker.*—I will say that the National Association took up this subject, there was a committee appointed for that purpose, and they have now invited every dental society in the United States to co-operate and assist them in the work.

*Dr. Boardman.*—Is there any further discussion? If not, I will ask Dr. Crockett if he has anything to say.

*Dr. Crockett.*—I do not think I have anything further to say, except that in my personal experience in examining the children, as I have said before, the parents made no objection whatever to it. But I found that it took a great deal of time. Of course, I took six or seven instruments of each kind. I could not possibly examine more than twenty children in two hours, and I had two of the teachers to assist me to take notes and get the children in line. It is an exceedingly difficult task to do it thoroughly.

On motion, adjourned.

H. W. Haley,
*Editor Harvard Odontological Society.*

